# Lost in Translation: Assessing Effectiveness of Focus Group Questioning Techniques to Develop Improved Translation of Terminology Used in HIV Prevention Clinical Trials

**DOI:** 10.1371/journal.pone.0073799

**Published:** 2013-09-10

**Authors:** Natasha Mack, Catalina B. Ramirez, Barbara Friedland, Soori Nnko

**Affiliations:** 1 Social and Behavioral Health Sciences, FHI 360, Durham, North Carolina, United States of America; 2 School of Medicine, University of North Carolina at Chapel Hill, Chapel Hill, North Carolina, United States of America; 3 HIV and AIDS Program, Population Council, New York, New York, United States of America; 4 National Institute for Medical Research, Mwanza, Tanzania; University of Washington, United States of America

## Abstract

**Introduction:**

Achieving participant comprehension has proven to be one of the most difficult, practical, and ethical challenges of HIV prevention clinical trials. It becomes even more challenging when local languages do not have equivalent scientific and technical vocabularies, rendering communication of scientific concepts in translated documents extremely difficult. Even when bilingual lexicons are developed, there is no guarantee that participants understand the terminology as translated.

**Methods:**

We conducted twelve focus groups with women of reproductive age in Mwanza, Tanzania to explore the effectiveness of four questioning techniques for: (1) assessing participants' familiarity with existing technical terms and concepts, (2) generating a list of acceptable technical and non-technical terms, (3) testing our definitions of technical terms, and (4) verifying participants' preferences for terms. Focus groups were transcribed, translated, and qualitatively analyzed.

**Results and Discussion:**

A translation process that uses all four questioning techniques in a step-wise approach is an effective way to establish a baseline understanding of participants' familiarity with research terms, to develop and test translatable definitions, and to identify participants' preferred terminology for international HIV clinical research. This may help to ensure that important concepts are not “lost in translation.” The results emphasize the importance of using a variety of techniques depending on the level of participant familiarity with research concepts, the existence of colloquial or technical terms in the target language, and the inherent complexity of the terms.

## Introduction

Achieving participant comprehension has proven to be one of the most difficult, practical, and ethical challenges of HIV prevention clinical trials. Language can be a key barrier to participant comprehension due to the use of technical terminology which may be unknown or unfamiliar to populations who participate in international clinical trials [1].

Achieving comprehension becomes even more challenging when local languages do not have equivalent scientific and technical vocabularies, rendering communication of scientific concepts in translated documents extremely difficult [2]–[7] and resulting in miscomprehension, inaccuracies, and the inadvertent introduction of cultural beliefs [3]. To a large degree, HIV prevention research has been conducted in Sub-Saharan Africa, where HIV prevalence is the highest. Kiswahili dialects serve as the lingua franca for 40 to 100 million people in the region [8]. However, despite the large number of speakers, Kiswahili, like many African languages, lacks scientific technical terminology in many topic areas, making translation of technical documents problematic [5], [9]–[12].

Although processes for translating documents in clinical trials are not standardized [13], [14], they usually consist of a variation of the Brislin method – a general practice of forward and back translation [15]. This method involves several rounds of independent forward and back translation of documents from the source language to the target language, back to the source language, and back again to the target language until a satisfactory rendition is achieved. The main advantage of the Brislin method is that the checks and balances inherent in the multiple rounds of forward and back translation ensure that semantic incongruences between the two versions of a document are identified and resolved; this reduces the potential for translation to introduce inaccuracies into a document.

To address the need for consistent translation of key terms across different types of study documents, some clinical trial researchers have begun to add a lexicon development step to the Brislin-derived translation process [9]. A lexicon is a content-specific dictionary consisting of an alphabetized list of terms and their definitions. In this process, researchers develop study-specific English lexicons prior to translating study documents and then collaborate with local translators to translate and back translate the research terms. Once researchers and translators agree upon the translations developed during the iterative forward- and back-translation process, the bilingual translations in the lexicon are used consistently across study documents, such as informed consent documents, study instruments, and participant information sheets.

Using bilingual lexicons may lead to the *consistent* translation of study documents; however, the critical disadvantage is that it relies on the linguistic expertise and cultural experience of the translator rather than of the trial participant. A translator may not always have a nuanced understanding of language commonly used by the study participants [4], or may have an educational level higher than that of the study population [3]. There may also be a variety of people serving in the role of translators, including investigators, study staff, and professional translation companies. Because the backgrounds of different translators will inevitably vary, systematic use of the Brislin method to develop a bilingual lexicon may still result in translations that include terms which are unfamiliar or not commonly used by the study population, impeding participant comprehension [16] [9], [11]. Therefore, even when clinical research studies utilize bilingual lexicons, the risk remains that participants could misinterpret or not comprehend key terms essential to the clinical trial process.

We conducted research to assess the effectiveness of four focus group questioning techniques derived from field linguistics and social science research methods [16]–[18] for developing and verifying lexicons for use in HIV prevention clinical trials. The process consisted of a series of focus groups with members of future clinical trial study populations. Here, we describe our analysis of whether the questioning techniques that we adapted for the focus groups were effective for:

- Learning participants' familiarity with existing technical terms
- Generating a list of technical and non-technical terms known to participants
- Testing our definitions of technical terms
- Learning participants' preferred translations

## Methods

### Ethics Statement

This study was conducted by FHI360 (USA) and the National Insitute for Medical Research (NIMR) (Tanzania) according to the principles expressed in the Declaration of Helsinki, and was approved by the Protection of Human Subjects Committee (PHSC) of FHI 360 and the Tanzanian National Health Research Ethics Review Sub-Committee (NatHREC). Written informed consent was obtained from all study participants.

### Study Design

We conducted two rounds of six focus groups with women from Mwanza, Tanzania in which we explored the effectiveness of four focus group questioning techniques in (1) assessing participants' familiarity with existing technical terms and concepts, (2) generating acceptable technical and non-technical terms, (3) testing our definitions of technical terms, and (4) verifying translation and term preferences.

### Study Population

Tanzanian women from Mwanza were recruited from bars, guesthouses and similar venues using eligibility criteria analogous to previous HIV prevention clinical trials conducted with this population. Women were eligible to participate if they were between the ages of 18 and 45, had never participated in a clinical trial, and reported at least one vaginal sex act in the previous 14 days or more than one sexual partner in the previous 30 days.

### Data Collection

Two rounds of data collection – the Development Phase and the Verification Phase – took place between October 2010 and May 2011. Six focus groups were conducted during each round of data collection. The number of focus groups was based on our assessment of what would be feasible to replicate in a clinical trial setting. Three questioning techniques were used in the first round, and a fourth technique was used in the second round only.

#### Development Phase

In the Development Phase, research staff created an English lexicon of technical terminology related to HIV prevention trials. The list was derived from lexicons from two HIV prevention trials [19], [20], as well as other guidance for the use of terminology in international HIV prevention research [21]. We selected 49 terms which we grouped into three broad topic areas: sexual behavior, reproductive health and infectious diseases, and research terms (see Table 1). For each term, we then developed English, plain-language definitions derived from the previous lexicons.

**Table 1:** Terms and questioning techniques, grouped by topic and phase.

TERM	TECHNIQUE			
	Term Elicitation	Term Explanation	Definition Explanation	Verbal Multiple Choice
Abstain/ Abstinent	R1			R2
Anal sex	R1			R2
Benefits	R1			R2
Bisexual man	R1			R2
Blood test	R1*			
Blood Draw	R1*			
Casual partner	R1			R2
Cervix	R1*			
Clinical trial		R1		R2
Concurrent sexual partner			R1	R2
Confidentiality	R1, R2			
Effectiveness	R1, R2			
Eligible	R1			R2
Enrol	R1			R2
Family planning method	R1			R2
Female sex partner	R1			R2
Genital wart/lesion	R1, R2			
HIV test	R1*			
HIV/AIDS	R1*			
Informed consent			R1	R2
Injectables	R1			
Intimate partner violence	R1, R2			
Male condom	R1*			
Male sex partner	R1			R2
Menstrual period	R1			R2
Microbicide	R1			R2
Monogamous	R1*			
Oral contraceptives	R1*			
Oral sex	R1			R2
Pap smear	R1			R2
Pelvic exam	R1			R2
Pre-exposure prophylaxis	R1			R2
Pregnancy test	R1*			
Pregnant	R1*			
Primary/Stable sexual partner	R1			R2
Randomization			R1	R2
Research study		R1		R2
Risks	R1			R2
Rounds [of sex]	R1			R2
Safe sex		R1		R2
Screening			R1	R2
Sexual intercourse	R1			R2
Sexually transmitted infection		R1		
Side effects	R1			R2
Speculum	R1			R2
Transactional sex	R1			R2
Urine sample/test	R1*			
Vaginal fluid	R1*			
Voluntary	R1			R2

* Term not included in Verification Phase.

A Tanzanian translator with expertise in translating research terminology was then identified. The local translator worked with local the FHI360/NIMR research team to generate translations of the lexicon terms and definitions in the dialect of Kiswahili spoken in Mwanza. For most words, the translator and Tanzanian principal investigator (PI) offered multiple translations that consisted of dictionary equivalents, words they considered most appropriate for a research context, and locally used words and expressions.

We developed three questioning techniques for use in the Development Phase focus groups. These techniques sought to (1) assess participants' familiarity with existing technical terms and concepts, (2) generate acceptable technical and non-technical terms, and (3) test our definitions of technical terms. Terms were matched with one or more techniques depending on the research objective (Table 1).

In cases where the local investigator and translator identified Kiswahili technical and non-technical equivalents, we used the Term Explanation technique, which is described below, to assess participants' familiarity with the suggested terms.

Term Explanation Technique: We asked participants to explain their understanding of translated terms to learn their familiarity with existing technical terms in Kiswahili. For example, for the term “sexually transmitted infection,” the following question and probes were developed:

*I'd like to ask about sexually transmitted infections, sometimes also called sexually transmitted diseases (STIs). What is a sexually transmitted infection?*

*Probes:*

- *How does a person become infected with a sexually transmitted infection?*
- *What are the different kinds of sexually transmitted infections?*
- *What are the symptoms of these infections?*

For terms that the translator and Tanzanian PI identified as familiar concepts and for which there were no known local-language equivalents, we developed adefinition and used the Term Elicitation Technique, described below, to test the definition and generate a list of technical and non-technical terms.

Term Elicitation Technique: We presented participants with definitions of terms and asked them to provide terms that matched the definition. For some questions, scenarios were used to provide a context for the definitions. The technique was used to simultaneously test our definitions and generate a list of technical and non-technical Kiswahili terms known to participants. For example, for the term “ female sex partner,” the following question and probes were developed:

*Next, I would like you to complete this sentence: When a man enters his penis into a woman's vagina, the woman is the man's ______________.*

*Probes:*

- *To whom would you be talking when you say [insert elicited term]?*
- *Does [insert elicited term] mean anything else? If so, what?*
- *Are there any other terms you would use when you speak to a doctor? Your husband or boyfriend? Your sister? Your friends?*

For terms, which were unfamiliar concepts **and** had no local language equivalents, we developed new definition and used the Definition Explanation Technique, described below, to test the definitions we created and generate a list of technical and non-technical terms.

Definition Explanation Technique: We provided an explanation of a term and then asked participants to explain it back to the group to learn whether our definition was easily understood. For example, we developed the following definition and question for the term “screening”:

*In most cases, study staff will ask the person some questions to find out if the person has all the required characteristics on the list. The person may also need to have some laboratory tests to find out about her health. For the research study you are participating in today, we asked you some questions to make sure you had all of the characteristics necessary. Could someone explain to the group what that process was like? What were the characteristics necessary to be in today's research?*

Focus groups were recorded and transcribed into Kiswahili by the moderators. The study translator, in consultation with the Tanzanian PI, then translated the Kiswahili transcripts into English. Researchers then reviewed the transcripts to assess whether participants understood the term or definition and to identify the elicited Kiswahili terms. We gauged comprehension of the term or definition by the similarity of terms elicited and participants' explanations of the term (Table 2).

**Table 2:** Criteria for evaluating effectiveness of the questioning techniques.

Technique	Objective	Effectiveness Criteria
**Term Explanation**	Assessing participants’ familiarity with existing technical terms	Were we able to ascertain, through an analysis of participant discussion, that participants were familiar with the technical/non-technical term?
		Did participants provide equivalent technical and non-technical terms?
		Did participants provide an explanation of the term that was technically correct?
**Term Elicitation**	Testing our definitions of technical terms and generating a list of technical and non-technical terms known to participants	Were we able to ascertain participants’ understanding of our translated definitions as well as participants’ familiarity with technical/non-technical terms?
		Were participants able to correctly answer questions about the definition content?
		Were participants able to correctly explain the term to the group after hearing the definition?
		Did participants provide equivalent technical and non-technical terms?
**Definition Explanation**	Testing our definitions of technical terms.	Were we able to ascertain participants’ understanding of our translated definitions as well as participants’ familiarity with technical/non-technical terms?
		Were participants able to correctly answer questions about the definition content?
		Were participants able to correctly explain the term to the group after hearing the definition?
**Verbal Multiple Choice**	Identifying participants’ preferred translations	Were we able to ascertain participants’ term preference?
		Did the questions get participants to identify a preferred term?
		Was there a consensus among the participants of the different focus group discussions?
		Did participants provide reasons for term preferences?

#### Verification Phase

In the Verification Phase, research staff sought to (1) determine participants' term preferences in cases where multiple term equivalents were elicited, and (2) assess participant familiarity with new term definitions in cases where the definition used in the Development Phase was unsuccessful.

For a subset of 13 terms, the techniques used in the Development Phase elicited a single term that was uniformly used by participants and matched the terms that the local translator and Tanzanian PI had suggested. These terms were not included in the Verification Phase (Table 1).

In four cases, techniques used in the Development Phase were re-employed in order to assess the new term definition. In this phase, we also used the fourth technique–Verbal Multiple Choice–for determining participants' term preferences. This technique, which is described below, was used for the 32 cases in which we elicited multiple technical and non technical terms.

Verbal Multiple Choice Technique: In order to identify term preferences, we asked participants to match our definition to the term that they liked best and that they felt most closely matched the original definition. We provided participants with term choices that included (1) words we had elicited in the Development Phase focus groups and (2) suggestions from the local translator and Tanzanian PI. Moderators read these choices aloud. We asked participants to vote on their favorite answer(s) and to explain the reasons for their selections. More than one answer was possible so that we could learn whether participants viewed multiple words as acceptable ways to communicate the definition. This technique was intended to learn participants' preferred terms in order to include them in the lexicon. For example, for the term “male sex partner,” the following question and probes were developed (translation notes included in brackets were not read to the participants):

*The counselor asks the research participant, Limi, if a man has inserted his penis into her vagina during the past seven days. I am going to give you four choices of words that the counselor could use to refer to this act. Listen to all of the choices first. Then when I read them again, close your eyes and raise your hand for the best choices of words. Which of the following four choices would the counselor most likely use to refer to this act?*

- *Kujamiiana [sexual intercourse – formal]*
- *Kufanya mapenzi [sexual intercourse, vaginal sex – word used in research – translator*'*s choice]*
- *Kutiana [sexual intercourse – informal]*
- *Kafanya tendo la ndoa [to have a sex act]*

*Probes:*

- *Why do you think [most voted answer] is the best choice for a counselor to use?*
- *Many of you also chose [next most voted answer]. Why do you think a counselor would use this word/phrase?*

Moderators transcribed and translated the Verification Phase focus group discussions and the translator then reviewed the English transcripts. In real time, moderators also filled out a data extraction form documenting how many people had voted for each Verbal Multiple Choice answer.

### Data Analysis

We developed criteria to evaluate the effectiveness of each questioning technique based on the objectives of each technique (Table 2), as the goal of data analysis was to evaluate the techniques' effectiveness rather than to conduct a thematic analysis. We then coded the transcripts using NVivo 9, a software program designed to assist in qualitative coding and analysis [22]. A two-step coding process was utilized. First, we applied codes representing the technique and specific term to each focus group transcript. We then individually analyzed segments of text and tagged them with codes that denoted the effectiveness of the technique (Table 2):

- We assessed the Term Explanation technique's effectiveness for indicating participants' familiarity with existing terminology based on whether the questions got participants to provide meaningful explanations of the terms and technical and non-technical term equivalents.
- We assessed the Definition Explanation and Term Elicitation techniques' effectiveness for eliciting a list of technical and non-technical terms known to participants and/or simultaneously evaluating the translatability of our definitions of terms based on whether the questions successfully generated equivalent terms and/or a correct explanation of the term from group members.
- We evaluated the Verbal Multiple Choice technique's effectiveness in allowing us to identify participants' preferred translations based on whether participants identified a preferred term and whether a consensus was reached among all participants.

To identify trends in effectiveness among the different questioning techniques, we generated and reviewed code frequency reports and coded text reports.

## Results

Sixty-one women participated in the 12 focus group discussions. There were an average of eight participants per focus group in the six focus groups of the first round –the Development Phase–for a total of 44 women, and an average of 10 women per focus group in the six focus groups of the second round–the Verification Phase.

Here, we describe the effectiveness of the four focus group questioning techniques, for use in developing and verifying lexicons for use in HIV prevention clinical trials, according to the criteria described in Table 2.

### Effectiveness in assessing participants' familiarity with existing technical terms and concepts

The Term Explanation technique was used with four of the 49 terms for which the translator identified existing Kiswahili technical and non-technical equivalents. The Term Explanation technique to gauge participants' familiarity with existing Kiswahili technical terms. In all four cases (i.e., “clinical trial,” “research,” “safe sex,” and “sexually transmitted infection/STI”), participants were familiar with the term, as indicated by their ability to provide detailed and technically correct explanations and identify equivalent technical and non-technical terms.

For the term “sexually transmitted infection,” for example, participants provided extensive examples of symptoms, including “raised bumps with pus,” “rashes,” and “pain during urination.” Participants were also able to identify several specific STIs, including HIV, gonorrhea and syphilis. For the term “safe sex,” they explained their understanding of the widely used Kiswahili translation *mapenzi salama* as: “to have sex without causing bruising or scratching,” “to trust each other,” “honesty,” and “using protection during sexual intercourse.” For the two more abstract terms – “research” and “clinical trial” – our questions included probes in addition to the explanation of the terms. The term “research,” for example, included the following explanation:

*Research is the process of carefully studying information to discover one or more facts. Let me give you an example. Scientists have developed a new drug. They want to know if it will cure malaria infection within one week. They must do research to learn the answer to this question. First they have to find 100 people with malaria who are willing to try the new drug. If it cures the malaria infections of most of the people within one week, scientists will learn the answer to the question.*

We also included the following two probes: *“Who does research? What is the purpose of research?”* Reviewing the focus group discussion in response to the question and probes allowed us to ascertain the depth of participants' understanding of each term. It was evident from participants' initial responses that many participants had been exposed to these terms/concepts, as when one participant indicated that “research is to look for something, I mean to investigate on something until you get the solution.”

### Effectiveness in generating a list of technical and non-technical terms and testing our definitions of technical terms

The Term Elicitation technique served the dual purposes of allowing us to test our definitions of technical terms identify equivalent technical and non-technical terms known to participants. Term Elicitation was used with 38 terms that the local investigator and translator identified as familiar concepts with no known Kiswahili technical or non-technical equivalents. In these cases, we translated the definitions and used the Term Elicitation technique to test the translated definition and generate a list of technical and non-technical terms. We assessed the technique's effectiveness by whether we were able to ascertain participants' understanding of our definitions and their familiarity with technical or non-technical terms (Table 2).

Overall, the Term Elicitation technique was used in 38 cases and was successful in all instances (38/38). Participants understood the definitions that were provided and during group discussion were able to identify complementary technical and non-technical terms. For example, as part of the discussion about the term “casual partner,” we provided a definition and several relationship scenarios and then asked participants to provide terms for the type of partner we had described. The discussion identified technical terms for a casual partner (*mpenzi wa kupita*), as well as local idioms and terms for this type of relationship such as *sukuma* (word for small tin, used to mean a secondary or casual partner), *kibotorwa* (word for small container, used to mean a secondary or causal partner), *mchicha* (a word for spinach which is used idiomatically to mean “secondary nourishment” or a casual partner).

Term Elicitation was also successful in indicating the lack of familiarity with a concept or a lack of equivalent terminology. In some cases, participants understood the definition they were provided but were not familiar with a corresponding term. For example, when provided with a picture and definition of genital warts, many women indicated that the picture showed a sexually transmitted infection. However, even though women were able to identify the ways in which genital warts differed from other STIs (i.e., raised bumps, warts) they were unable to provide a term that referred specific to this condition.” Even after a refinement of the definition for the Verification Phase, focus groups yielded similar results.

In other cases we defined procedures, objects, behaviors, or conditions which lacked equivalent terms in Kiswahili, such as “transactional sex,” “concurrent sexual partners,” “genital wart,” “pap smear,” and “pelvic exam.” The discussion indicated that participants understood the definition we provided but were not familiar with technical or non-technical terms to represent the concept. For some terms, such as “pap smear,” participants had no experiential frame of reference by which to identify equivalent terms. Participants had never undergone a pap smear, pelvic exam, or any similar gynecologic exam, thus they provided general terms such as “intensive check-up,” “pregnancy check-up,” or “cervix cleaning” that were based on their knowledge of medical procedures.

Term Elicitation was also successful in indicating when participants *did not* understand the definitions we provided. For the terms “confidentiality” and “effectiveness,” a technical definition was provided to participants and they were asked to provide an explanation and similar terms. The discussion indicated that participants only partially understood the definition, thus making it impossible to ascertain participants' familiarity with the concept. In the case of the term “effectiveness,” participants provided explanations such as “the medicine has been taken,” which did not include discussion of the extent to which a drug worked to prevent or treat a medical condition. Based on participant discussion, we then created a new definition that included a scenario in which a malaria treatment drug was tested and only found to cure a portion of the people who contracted the disease. The inclusion of a scenario with a familiar example facilitated participant discussion and allowed us to ascertain participants' understanding of the new definition as well as their familiarity with the technical concept and similar terms. Participants were able to produce non-technical phrases and descriptions to explain the concept, such as “successfulness of a drug,” “the drug treats,” and “the drug is reliable.”

We used the Definition Explanation technique with four of the 49 terms, which the local investigator and translator identified as unfamiliar concepts. For these terms we developed definitions and used the definition explanation technique to simultaneously test the definition. We assessed the technique's effectiveness by whether we were able to ascertain participants' understanding of our definitions (Table 2).

Overall, Definition Explanation was successful in all four cases. In two cases, “informed consent” and “screening,” participants were able to provide detailed explanations of the concepts, thus allowing us to ascertain their understanding of the definition. The definitions for these terms referenced participants' own experience enrolling in our study and gave participants a point of reference for their discussion of the concepts.

In the other two cases, “concurrent sexual partners” and “randomization,” the technique was effective in ascertaining that participants did not understand the definition we provided. When given a definition of the term “randomization,” participants had difficulty understanding how “random” assignment would take place, saying that they would “take a side,” “be appointed,” or “be selected.” In the case of “concurrent sexual partnerships,” participant discussion indicated that participants did not distinguish nuances in the definition related to partnership overlap. Participants provided terms that were used to identify someone involved in multiple, although not necessarily concurrent, sexual partnerships such as “prostitute,” “sex swindler,” and “player”

### Effectiveness in identifying participants' preferred translations

The Verbal Multiple Choice technique was used exclusively in the Verification Phase to learn participants' preferences for terms. We evaluated the Verbal Multiple Choice technique's effectiveness based on whether participants were able to identify a term they preferred and on whether we were able to come to a consensus on a preferred term to use in the lexicon. We assigned the cutoff for a “consensus term” as a term that garnered more than half of the total votes across all focus groups and was at least 10 percentage points higher than the “runner up.” This cutoff was determined by reviewing the natural spread of points in the data. The Verbal Multiple Choice technique was used for 32 terms for which we had elicited multiple technical and non technical terms in the development phase; however, the responses for one term (“pap smear”) had to be discarded from the analysis due to incorrect translation of the question.

We found the technique to be overwhelmingly effective (28 of 31) for learning which terms participants preferred and for identifying instances in which more than one term was acceptable. Of the 28 cases in which the Verbal Multiple Choice technique worked, we found that there was a consensus on a preferred term in 24 cases. In some instances, terms elicited in the Development Phase were unpopular with the participants in the Verification Phase. For example, a picture of a speculum had as its choices: a technical translation, “duck's mouth” which had been suggested and agreed upon by participants in the Development Phase focus groups, and “cervix opener/expander,” a descriptive term the moderator had spontaneously proposed in the Development Phase focus groups to try to describe the unfamiliar object. Only two of the 31 participants chose “duck's mouth,” with the most popular answer being “cervix opener/expander.”

Although successful for identifying term preferences, the Verbal Multiple Choice technique was only moderately effective for learning why participants preferred certain terms. Some questions yielded useful information on usage, including which terms would be most widely understood, which terms were vulgar or not appropriate, and to some extent why some terms were not appropriate choices. However, for more than half of the terms, participants could not articulate why they felt a particular response worked better than another. For example, for the term “confidentiality,” almost three quarters of participants preferred the term “confidential,” whereas all others selected the term “privately.” In both cases, participants cited that their preferred term meant that individuals “do not share facts or details with anyone else” and “keep information secret.” However, when participants were specifically asked why the term that they had not selected was less appropriate, participants once again cited the reasons for their selection, rather than a rationale for its appropriateness.

Additionally, the Verbal Multiple Choice technique was ineffective in three cases (i.e., “concurrent sexual partners,” “randomization,” and “rounds of sex”). In these cases, we attempted to use a definition that had been revised following the Development Phase but not assessed using the Definition Explanation or Term Elicitation techniques. In all three cases, analysis of the discussion indicated that the participants did not understand the revised definition, and thus we were unable to ascertain term preferences.

## Discussion

Regions of the world that bear the most disproportionate burden of infection, especially Sub-Saharan Africa, are frequently the context for HIV prevention trials. However, the successful and ethical conduct of these clinical trials rests on the ability of trial participants to clearly understand what they are being asked to do.

In this analysis, we sought to evaluate the effectiveness of four questioning techniques for gauging participants' familiarity with existing terms, generating a list of technical and non-technical terms known to participants, testing definitions of terms, and learning participants' preferences for translated terms. Our findings indicate that a translation process that uses all four questioning techniques in a step-wise approach is an effective way to establish a baseline understanding of participants' familiarity with research terms; develop and test translatable definitions; and identify preferred terminology for HIV clinical research. This could help ensure that concepts critical to participants' understanding of clinical trial procedures and their involvement in clinical trials are not “lost in translation.”

**Figure 1:**
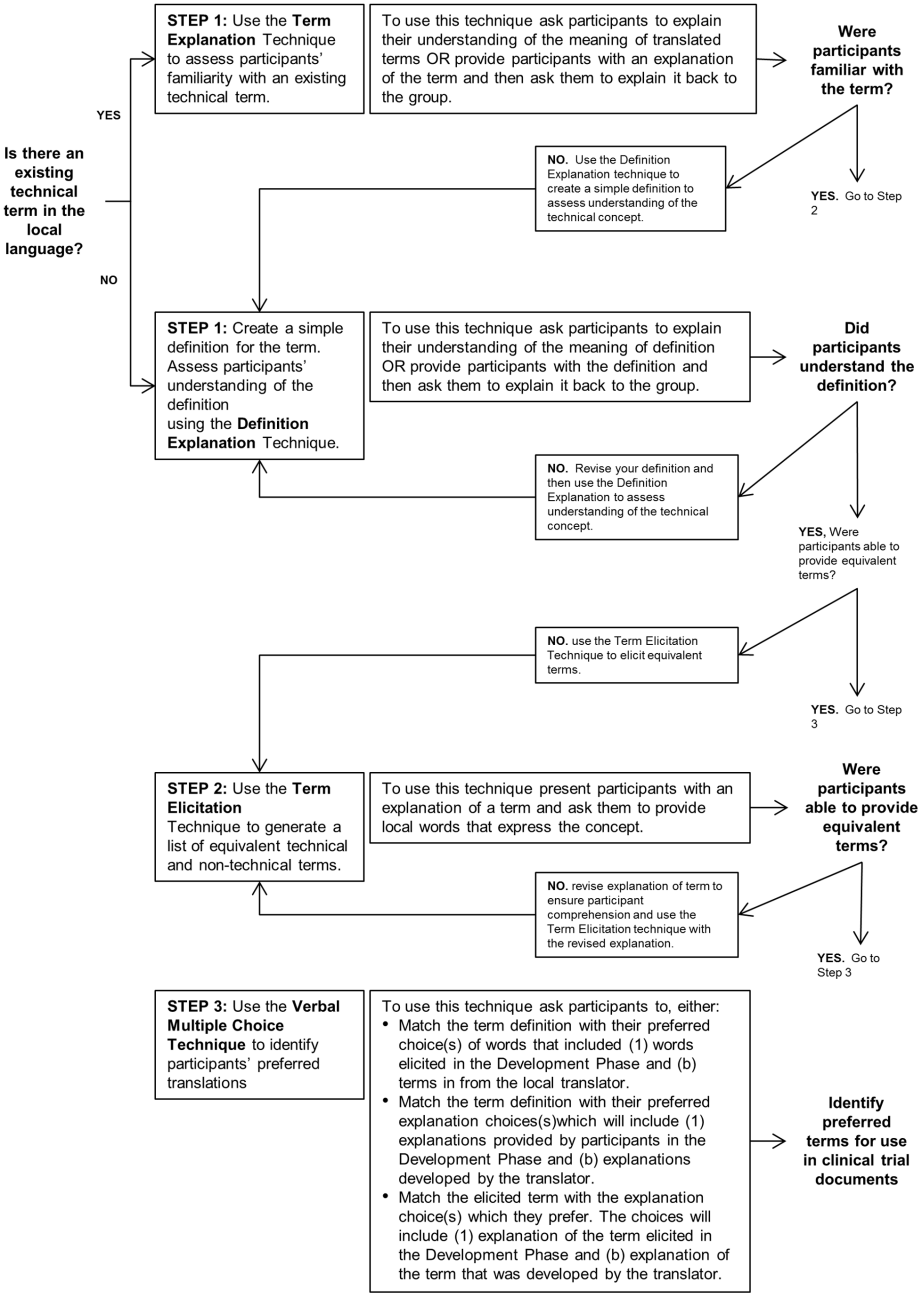
Recommended Guidelines for Using Techniques.

Furthermore, the results emphasize the importance of using a variety of techniques depending on the level of participant familiarity with research concepts, the existence of colloquial or technical terms in the target language, and the inherent complexity of the terms. The step-wise process we recommend is outlined below and diagrammed in Figure 1.

### STEP 1: Gauge participant understanding of a term or existing definition

Definition Explanation was an effective first step for gauging participants understanding of a concept or plain-language definition. However, when participants were not able to explain the definition as part of this technique, it fell to the moderator to probe spontaneously in order to determine whether this was due to an inadequate definition or to participants' lack of familiarity with the concept. Therefore, focus group moderators need to be prepared for this possibility. Additionally, our findings suggest that even in cases where an existing technical term is thought to be well known among the study population, it is best to verify that participants are familiar with the term using Term Explanation prior to using it in a clinical trial setting.

### STEP 2: Identify technical or non-technical terms that could be used to communicate the concept

Definition Explanation and Term Elicitation were both effective ways to elicit technical and non-technical terms that could be used to communicate a concept. Our results indicate that scenarios that referenced participants' own experiences enhanced the effectiveness of both of these techniques. We would also note that previous studies have highlighted participants' preferences for non-technical terminology [23]–[27].

### STEP 3: Identify participants' preferred terms

As a final verification step, the Verbal Multiple Choice technique should be used to ascertain participants' word preferences and identify cases in which multiple terms are acceptable. Even though only one term might be used when applying the lexicon to clinical trial documents, including multiple verified terms as part of study staff training or staff scripts and job aids may help facilitate participant comprehension.

### Limitations

Our study was preliminary in that it did not include an evaluation component that would have allowed us to determine whether the preferred translated terms identified by the study population, as well as our plain-language definitions for words that have no technical equivalent, led to improved comprehension when used in informed consent forms or other study materials. A critical next step is to assess participants' comprehension of informed consent forms that incorporate terminology and definitions elicited and/or verified in focus groups and compare them with participants' comprehension of informed consent forms prepared via the standard forward translation – back translation process. An assessment of the readability of the consent forms should also be part of this next research step.

The four questioning technique were evaluated with terms that are specific to HIV prevention research, but we believe that our findings may also apply to translation of materials used in other types of international clinical trials. Materials could include informed consent forms, but also quantitative data collection forms, qualitative instruments, study product adherence counseling, and community education.

In addition, the data collection methods we used were specifically chosen in an effort to identify processes for lexicon development within the context of HIV prevention clinical trials. Thus, this study sought to develop a process that could be integrated into existing research activities at clinical trial sites. Cross-cultural adaptation techniques, including cognitive interviewing and committee-based translation, which are utilized in other research settings for adapting study instruments, for example, may be able to address some of the limitations of the techniques described here, and should be explored.

## References

[1] Waggoner WC, Mayo DM (1995) Who understands? A survey of 25 words or phrases commonly used in proposed clinical research consent forms. IRB 17: 6–9. 11653061

[2] Elbourne D, Snowdon C, Garcia J (1997) Informed consent. Subjects may not understand concept of clinical trials. BMJ 315: 248–249. PMC2127151 9253281

[3] Marshall PA (2006) Informed consent in international health research. J Empir Res Hum Res Ethics 1: 25–42. 19385865 10.1525/jer.2006.1.1.25

[4] Woodsong C, Karim QA (2005) A model designed to enhance informed consent: experiences from the HIV Prevention Trials Network. Am J Public Health 95: 412–419. 15727968 10.2105/AJPH.2004.041624 PMC1449193

[5] Tekola F, Bull SJ, Farsides B, Newport MJ, Adeyemo A, et al (2009) Tailoring consent to context: designing an appropriate consent process for a biomedical study in a low income setting. PLoS Negl Trop Dis 3: e482. 19621067 10.1371/journal.pntd.0000482 PMC2705797

[6] Bhutta ZA (2004) Beyond informed consent. Bull World Health Organ 82: 771–777. 15643799 PMC2623030

[7] Lawson SL, Adamson HM (1995) Informed consent readability: subject understanding of 15 common consent form phrases. IRB 17: 16–19. 11653358

[8] Kula NC, Marten L, editors (2008) Central, East and Southern African Languages. Berkeley and Los Angeles: Ivy Press/University of California Press. 111 p.

[9] Molyneux CS, Peshu N, Marsh K (2004) Understanding of informed consent in a low-income setting: three case studies from the Kenyan Coast. Soc Sci Med 59: 2547–2559. 15474208 10.1016/j.socscimed.2004.03.037

[10] Kass NE, Hyder A (2001) Attitudes and Experiences of U.S. and Developing Country Investigators Regarding U.S. Human Subjects Regulations. Bethesda, MD: National Bioethics Advisory Commission.

[11] Penn C, Evans M (2009) Recommendations for communication to enhance informed consent and enrolment at multilingual research sites. African Journal of AIDS Research 8: 285–294. 25864544 10.2989/AJAR.2009.8.3.5.926

[12] Krosin MT, Klitzman R, Levin B, Cheng J, Ranney ML (2006) Problems in comprehension of informed consent in rural and peri-urban Mali, West Africa. Clin Trials 3: 306–313. 16895047 10.1191/1740774506cn150oa

[13] Maneesriwongul W, Dixon JK (2004) Instrument translation process: a methods review. J Adv Nurs 48: 175–186. 15369498 10.1111/j.1365-2648.2004.03185.x

[14] Penn C, Evans M (2010) Assessing the impact of a modified informed consent process in a South African HIV/AIDS research trial. Patient Educ Couns 80: 191–199. 19963332 10.1016/j.pec.2009.10.019

[15] Brislin RW (1970) Back-Translation for Cross-Cultural Research. Journal of Cross-Cultural Psychology 1: 185–216.

[16] Weeks A, Swerissen H, Belfrage J (2007) Issues, challenges, and solutions in translating study instruments. Eval Rev 31: 153–165. 17356181 10.1177/0193841X06294184

[17] Shore S, van den Berg R (2006) A new mass elicitation technique: the dictionary development program. Paper presented at Tenth International Conference on Austronesian Linguistics. 17–20 January 2006. Puerto Princesa City, Palawan, Philippines. Available: http://www.sil.org/asia/philippines/ical/papers.html. Accessed 2013 Aug 16.

[18] Jones PS, Lee JW, Phillips LR, Zhang XE, Jaceldo KB (2001) An adaptation of Brislin's translation model for cross-cultural research. Nurs Res 50: 300–304. 11570715 10.1097/00006199-200109000-00008

[19] Skoler-Karpoff S, Ramjee G, Ahmed K, Altini L, Plagianos MG, et al (2008) Efficacy of Carraguard for prevention of HIV infection in women in South Africa: a randomised, double-blind, placebo-controlled trial. The Lancet 372: 1977–1987. 10.1016/S0140-6736(08)61842-5 19059048

[20] Van Damme L, Corneli A, Ahmed K, Agot K, Lombaard J, et al (2012) Preexposure Prophylaxis for HIV Infection among African Women. New England Journal of Medicine 367: 411–422. 22784040 10.1056/NEJMoa1202614 PMC3687217

[21] Joint United Nations Programme on HIV/AIDS (2011) UNAIDS terminology guidelines. Geneva: Joint United Nations Programme on HIV/AIDS.

[22] QSR International Pty Ltd. (2010) NVivo qualitative data analysis software, Version 9.

[23] Davis TC, Holcombe RF, Berkel HJ, Pramanik S, Divers SG (1998) Informed consent for clinical trials: a comparative study of standard versus simplified forms. J Natl Cancer Inst 90: 668–674. 9586663 10.1093/jnci/90.9.668

[24] Jhanwar VG, Bishnoi RJ (2010) Comprehensibility of translated informed consent documents used in clinical research in psychiatry. Indian J Psychol Med 32: 7–12. 21799552 10.4103/0253-7176.70517 PMC3137819

[25] LoVerde ME, Prochazka AV, Byyny RL (1989) Research consent forms: continued unreadability and increasing length. J Gen Intern Med 4: 410–412. 2795264 10.1007/BF02599693

[26] Meade CD, Howser DM (1992) Consent forms: how to determine and improve their readability. Oncol Nurs Forum 19: 1523–1528. 1461766

[27] Ridpath JR, Wiese CJ, Greene SM (2009) Looking at research consent forms through a participant-centered lens: the PRISM readability toolkit. Am J Health Promot 23: 371–375. 19601476 10.4278/ajhp.080613-CIT-94

